# Co-designing a Lifestyle-Focused Text Message Intervention for Women After Breast Cancer Treatment: Mixed Methods Study

**DOI:** 10.2196/27076

**Published:** 2021-06-14

**Authors:** Anna Singleton, Rebecca Raeside, Stephanie R Partridge, Molly Hayes, Katherine Maka, Karice K Hyun, Aravinda Thiagalingam, Clara K Chow, Kerry A Sherman, Elisabeth Elder, Julie Redfern

**Affiliations:** 1 Consumer Engagement and Codesign Research Hub, School of Health Sciences Faculty of Medicine and Health University of Sydney Sydney Australia; 2 Westmead Applied Research Centre Faculty of Medicine and Health University of Sydney Sydney Australia; 3 Prevention Research Collaboration, Charles Perkins Centre University of Sydney Sydney Australia; 4 Department of Physiotherapy Westmead Hospital Sydney Australia; 5 Westmead Breast Cancer Institute Westmead Hospital Sydney Australia; 6 Department of Cardiology Concord Repatriation General Hospital Sydney Australia; 7 Department of Cardiology Westmead Hospital Sydney Australia; 8 George Institute for Global Health University of New South Wales Sydney Australia; 9 Centre for Emotional Health Department of Psychology Macquarie University Sydney Australia; 10 Research Education Network Western Sydney Local Health District Sydney Australia

**Keywords:** breast neoplasms, cancer survivors, text messaging, telemedicine, mobile health, co-design

## Abstract

**Background:**

Breast cancer is the most common cancer among women globally. Recovery from breast cancer treatment can be mentally and physically challenging. SMS text message programs offer a novel way to provide health information and support, but few programs are co-designed with consumer representatives.

**Objective:**

This study aims to report the procedures and outcomes of a co-design process of a lifestyle-focused SMS text message program to support women’s mental and physical health after breast cancer treatment.

**Methods:**

We followed an iterative mixed methods two-step process: (1) co-design workshop with consumers and health professionals and researchers to draft text messages and (2) evaluation of message content, which was scored (5-point Likert scale; 1=strongly disagree to 5=strongly agree) for ease of understanding, usefulness, and appropriateness, and readability (Flesch-Kincaid score). Additional free-text responses and semistructured interviews were coded into themes. Messages were edited or deleted based on the evaluations, with consumers’ evaluations prioritized.

**Results:**

In step 1, co-designed text messages (N=189) were semipersonalized, and the main content themes were (1) physical activity and healthy eating, (2) medications and side effects, (3) mental health, and (4) general breast cancer information. In step 2, consumers (n=14) and health professionals and researchers (n=14) provided 870 reviews of 189 messages and found that most messages were easy to understand (799/870, 91.8%), useful (746/870, 85.7%), and appropriate (732/870, 84.1%). However, consumers rated 50 messages differently from health professionals and researchers. On the basis of evaluations, 37.6% (71/189) of messages were deleted, 36.5% (69/189) were edited, and 12 new messages related to fatigue, self-care, and cognition were created. The final 130 text messages had a mean 7.12 (SD 2.8) Flesch-Kincaid grade level and 68.9 (SD 15.5) ease-of-reading score, which represents *standard* reading ease.

**Conclusions:**

Co-designing and evaluating a bank of evidence-based mental and physical health-themed text messages with breast cancer survivors, health professionals, and researchers was feasible and resulted in a bank of 130 text messages evaluated highly by participants. Some consumer evaluations differed from health professionals and researchers, supporting the importance of co-design.

## Introduction

### Background

Breast cancer is the most commonly diagnosed cancer among women globally [1]. More than 2 million women finish active breast cancer treatment (ie, surgery, chemotherapy, or radiotherapy) yearly [1], and many experience treatment-related mental and physical health challenges [2,3]. The 2013 Breast Health Global Initiative consensus statement posits that access to posttreatment information and support should be allocated to all patients [3]. However, there are limited survivorship care services [3]. Existing services usually occur in person, during work hours, or far from home, which increases the financial burden [4] and limits accessibility. Moreover, these services are rarely co-designed with patients [5,6]. Accessible posttreatment health support programs are required.

### The Importance of Consumer Co-design

Co-design is the process by which service providers and consumers collaborate to develop meaningful and creative solutions. The benefits of co-design include improved services, provider-consumer interactions, and consumer engagement and experiences [7]. The key to effective co-design is making end-users’ or consumers’ lived experiences central throughout the project’s lifespan [8] because it adds a unique and diverse perspective [8,9]. For example, *citizen collaborators* can be employed as active members of the research team from study conception to final dissemination [9]. Qualitative data suggest that access to posttreatment health information and support are among breast cancer survivors’ top priorities for service improvement [2,10]. Enlisting breast cancer survivors as co-designers for novel posttreatment services may help place survivors’ priorities at the forefront and strengthen the intervention’s impact.

### Co-design and Mobile Health Interventions

Mobile health (*mHealth*) uses mobile devices such as mobile phones to deliver health services [11]. mHealth interventions delivered by text messages are the most accessible to patients, as they are inexpensive, do not require internet for delivery, and can be sent anytime and anywhere with mobile services [12]. Moreover, text message programs effectively deliver health education to patients [12,13] and are easily co-designed [14]. Co-designed text message programs improved health-promoting behaviors among patients with heart disease [13] and were cost-saving for the health care system compared with traditional delivery methods [15]. A 2015 systematic review revealed that there were no text message interventions for cancer survivors [16]; more recently, there is some evidence that breast cancer survivors find the delivery of health support via text messages acceptable [17,18] and effective for improving adherence to endocrine therapy tablets [19,20] or maintain weight loss [21]. However, no studies reported that the interventions were co-designed and most focused on physical health outcomes, not including mental health messages within the intervention. Therefore, co-designing a text message intervention with women who completed active breast cancer treatment may be an innovative solution to reduce barriers to posttreatment health information and support.

### Objectives

This study aims to report the procedures and outcomes of a co-design process of a bank of evidence-based text messages to support women’s mental and physical health after breast cancer treatment. The co-designers were women who completed active breast cancer treatment (consumers and citizen collaborators), health professionals, and researchers. We hypothesize that consumers and citizen collaborators would add value by providing different inputs and evaluations regarding lived experience with breast cancer and the health care system compared with health professionals and researchers.

## Methods

### Study Design

**Figure 1:**
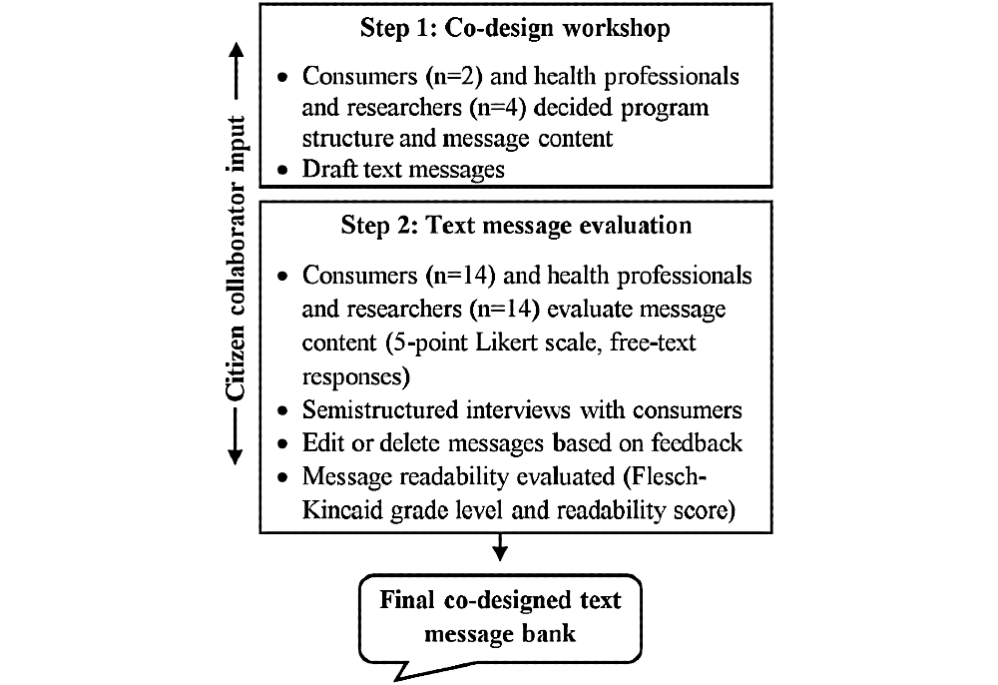
Study design, including a citizen collaborator throughout.

A two-step mixed methods process [14] was used, including consumer representatives and a citizen collaborator (MH) as co-designers (Figure 1). Briefly, (step 1) an initial co-design workshop and (step 2) a structured qualitative text message evaluation and program refinement were conducted from June 2018 to March 2019 at the Westmead Breast Cancer Institute (WBCI), a major public breast cancer institute in New South Wales. WBCI resides within a large tertiary hospital in Western Sydney, Australia, serving a multicultural and diverse socioeconomic population (approximately 2.3 million people in 2016) [22]. The study was approved by the Western Sydney Local Health District Human Research Ethics Committee (AU RED HREC/18/WMEAD/281). All procedures performed in studies involving human participants were in accordance with the ethical standards of the institutional research committee and with the 1964 Helsinki declaration and its later amendments or comparable ethical standards. Written or electronic informed consent was obtained from all the participants.

### Step 1: Co-design Workshop

The aim of step 1 was to work with consumer representatives, a citizen collaborator, health professionals, and researchers to develop the SMS text message program structure, content themes, and draft text message content.

#### Participants and Recruitment

Consumers were included if they had completed active breast cancer treatment (surgery, chemotherapy, or radiotherapy) within the past 5 years and did not have distant metastatic disease. Health professionals and researchers were eligible to participate if they were practicing health professionals (eg, medicine, allied health, or nursing) or active researchers with expertise in breast cancer or text message interventions. Consumers (n=3) from a local volunteer association, health professionals (n=8) from WBCI, and researchers (n=5) from 2 universities in Sydney, Australia, were invited via email to participate in a two-hour workshop to develop the program structure and message content. Those who agreed to attend were provided with further instructions regarding the workshop location. A citizen collaborator also reviewed the program structure and developed the message content. The citizen collaborator was aged 29 years, a White woman with lived experience with breast lumpectomy surgery, chemotherapy, radiotherapy, and 1 year of taking endocrine therapy tablets. The citizen collaborator assisted in developing the research idea and provided ongoing feedback throughout the program design.

#### Co-design Workshop

A two-hour interactive workshop was held on June 5, 2018. The workshop included a 15-minute presentation and semistructured discussion topics, such as the number of text messages delivered per week, if the delivery was one-way (no replies) or two-way (replies allowed), and message content themes. Time was also allocated to draft text message content. After the workshop, the health professionals drafted additional messages. The program structure and message content were discussed with the citizen collaborator before and after the workshop.

#### Theoretical Basis for Message Content

The text messages were developed based on the behavior change techniques taxonomy by Abraham and Michie [23], which includes the Bandura theory of social learning theory (social cognitive theory) and the information-motivation-behavioral skills model. The theories stipulate that providing information through education and motivation and observing others successfully complete behaviors can promote behavior change [24,25]. Moreover, assisting people in setting attainable goals and identifying barriers and strategies for overcoming barriers can facilitate goal achievement [24]. Table 1 provides examples of message content and corresponding behavior change techniques. In addition, researchers have found that people enjoy supportive personalized messages with a positive tone [26,27]. The messages were co-designed with these user satisfaction principles in mind, including achievable, practical tips and links to trustworthy websites and videos.

**Table 1:** Example of draft text message content and themes with corresponding behavior change techniques.

Behavior change technique	Example text message content	Message theme
Provide information about behavior-health link (IMB^a^)	Fibre in fruit, veggies and wholegrain foods helps you feel full for longer, can improve blood sugars & lower cholesterol - so make sure there’s plenty of fibre in your diet!	Nutrition
Provide information on consequences (TRA^b^, TPB^c^, SCogT^d^, or IMB)	Research shows that consistent physical activity can help reduce antihormonal (endocrine) tablet side effects - so try to get moving every day but make sure to schedule time to rest!	Medication adherence and side effects
Prompt intention formation (TRA, TPB, SCogT, or IMB)	Sometimes we can do exercise without noticing - challenge yourself to park the car further away from the shops or your work so you get a few extra steps!	Physical activity
Prompt barrier identification (SCogT)	Side effects are the main reason why women stop taking their antihormonal (endocrine) tablets - Remember, [pref_name]^e^, your doctor has prescribed this medication to benefit your health and can help you if side effects are a problem.	Medication adherence or side effects
Provide general encouragement (SCogT)	Hi [pref_name], you are halfway through the EMPOWER-SMS^f^ program – it’s wonderful that you’re taking a few moments each week to focus on your health and wellbeing - keep it up!	General
Set graded tasks (SCogT)	Sometimes getting started is the hardest part, [pref_name] - it can be easier to begin exercise at low intensity (walking, stretching) and gradually increase to moderate intensity (faster breathing rate but can still have a conversation)	Physical activity
Provide instruction (SCogT)	Physical activity is essential for your recovery - World Health Organization recommends at least 30mins of moderate-intensity activity 5days/week - click here for ideas: [insert link here]	Physical activity
Model or demonstrate the behavior (SCogT)	Some women experience tightness or restricted movement in their shoulder long after treatments have finished - if this is you, the BCI^g^ shoulder care video may help: [insert link here]	Physical activity
Prompt specific goal setting (CT^h^)	Hi [pref_name], relaxation and self-care are important - try making a list of 3 things that help you relax so you can include them into your week	Social or emotional well-being
Prompt self-monitoring of behavior	Do you have trouble sleeping? Consider keeping a sleep diary, it may help you understand what’s impacting your sleep, like TV, lights or stress - see an example here: [insert link here]	General
Provide contingent rewards (OC^i^)	Did you take your antihormonal (endocrine) tablet today, [pref_name]? If yes, well done! If not, don’t worry, just take it as soon as you remember - if it’s nearly time to take your next tablet, do not take two at the same time - 1 per day is enough	Medication adherence or side effects
Teach to use prompts or cues (OC)	Usually, people serve too much food on a large plate - reducing your plate size can help limit portion sizes and avoid overeating! - [centre_name]	Nutrition
Prompt practice (OC)	Writing a list of 3 things you’re grateful for each week may change the way you feel – it’s also a nice way to reflect - [centre_name]	Social or emotional well-being
Provide opportunities for social comparison (SCompT)	Hi [pref_name], having a hard time talking about your breast cancer? Breast Cancer Network Australia has a great web-based forum, where you can read women’s questions and concerns, and you can pose questions, if you want. Here’s the link: onlinenetwork.bcna.org.au	Social or emotional well-being
Plan social support or social change (social support theories)	Exercising with a friend can be fun & you can motivate each other - grab a friend and get moving or meet some new friends at a local walking group, find one near you here: [insert link here]	Physical activity
Prompt self-talk	Practicing positive self-talk is a good way to keep your mind healthy and improve your mood - not sure where to start? Click for more information: [insert link here]	Social or emotional well-being
Relapse prevention	Making small changes can make a big difference when it comes to healthy eating - at restaurants look for simple switches like a baked potato instead of chips. Read more tips here: [insert link here]	Nutrition
Stress management (stress theories)	Mindfulness meditation can help to manage stress and improve mood by focusing on breathing and relaxation techniques - if you’d like to learn more, click here: [insert link here]	Social or emotional well-being
Motivational interviewing	Remember to be kind to yourself, [pref_name]! Think of 3 things you’re thankful that your body can do	General
Time management	Treat yourself to some *you* time, [pref_name] - whether it’s a walk, yoga or reading a good book, setting aside time in your diary or on your phone can make it easier to stick to	Social or emotional well-being

^a^IMB: information-motivation-behavioral skills model.

^b^TRA: theory of reasoned action.

^c^TPB: theory of planned behavior.

^d^SCogT: social cognitive theory.

^e^[pref_name] is a place holder for the auto-population of the participant’s preferred name.

^f^EMPOWER-SMS: A text message intervention to support women’s mental and physical health after breast cancer treatment.

^g^BCI: breast cancer institute.

^h^CT: control theory.

^i^OC: operant conditioning.

#### Medical Content of Messages

Medical content was based on personal experiences of health professionals and confirmed using Cancer Australia’s national guidelines for primary prevention of cancer [28], managing menopausal symptoms after breast cancer treatment [29], and emerging breast cancer survivorship literature and systematic reviews.

The authors reviewed all messages (AS, JR, SRP, and RR) before text message evaluation and refinement (step 2). The reasons for deletions were recorded.

### Step 2: Text Message Evaluation and Refinement

The aim of step 2 was for consumers, a citizen collaborator, health professionals, and researchers to evaluate the quality of the text message content, refine the messages based on feedback, and evaluate the messages’ ease of reading.

#### Participants and Recruitment

Consumers (n=16), the citizen collaborator, and health professionals and researchers (n=14) from the WBCI and 2 universities were invited to participate. Consumers were recruited during a medical clinic for one-year postsurgery follow-up appointments. Consumers were approached by their physician if they were an adult (>18 years) woman and completed active breast cancer treatment within the past 3 years (could still be taking endocrine therapy treatment) and were able to provide written informed consent. Consumers were not approached if they had metastatic breast cancer or insufficient English skills to provide informed consent or read and evaluate text messages.

Health professionals and researchers were eligible to participate if they were practicing health professionals (eg, medicine, allied health, or nursing) or active researchers with expertise in breast cancer or text message interventions. Health professionals and researchers known to the research team were invited to participate via email and provided electronic consent via a web-based database.

#### Text Message Evaluation by Consumers and Health Professionals

A combination of feedback surveys and semistructured interviews were conducted to evaluate the message content. For the feedback surveys, each participant provided basic demographic information regarding age, sex, and expertise (lived experience or health professional or researcher), and then evaluated 32 draft text messages (maximum 15 min) presented as 1 to 2 sentences on paper in a private room (consumers) or via a password-protected web-based database (health professionals or researchers). Owing to time constraints, one health professional evaluated only 16 messages. Message content related to medication adherence and side effects were reviewed by consumers who reported taking endocrine therapy tablets previously or at the time of the study.

The feedback survey contained 4 questions for each draft text message, with three 5-point Likert scale (1=strongly disagree to 5=strongly agree) questions: “This message was easy to understand;” “The information provided in this message is useful;” “This message is appropriate for women with breast cancer” and one free-text response question, “Do you have any suggestions to improve this message?” The Likert scale questions were summarized by the number and percentage of consumers’ or health professionals and researchers’ evaluations that *agreed* or *strongly agreed* that the messages were easy to understand, useful, and appropriate. Internal consistency was found among the 3 questions (Cronbach α=.88); therefore, each message received a mean score out of 15 (5 points per question; *strongly disagree*=1 point, *strongly agree*=5 points), which was the total score provided by each participant divided by the number of participants that evaluated the message. The mean consumer versus health professional and researcher score was also calculated for each message. Researchers were guided by a mean score <12 out of 15 (<80%), indicating less preference for a message from participants. All message scores and free-text replies were reviewed in parallel to guide message edits or deletions. If consumers and health professionals or researchers’ ratings differed, priority was given to consumers’ evaluations.

Free-text consumer and health professional or researcher evaluations were collated for each message. The citizen collaborator evaluated all messages via free-text feedback. After completing the feedback survey, consumers were invited to brief (10-15 min) semistructured interviews that explored participants’ most and least liked text messages, suggestions for additional message themes, the appropriateness of the language within the messages (too complicated vs too simple), and whether a SMS text message program would have been helpful during the transition from hospital care to health self-management. The interviews were audio-recorded and transcribed verbatim. The free-text feedback and interview transcripts were open-coded using inductive thematic content analysis in NVivo 11 (QSR International) by 2 independent parallel coders (AS and RR). The researchers discussed codes until they reached an agreement on themes. The feedback survey results and interviews were used to inform message refinement (edits, deletions, or creation of new message content).

#### Evaluation of Readability

All messages were evaluated for readability using the Flesch-Kincaid ease-of-reading score and grade level, which are validated scores of content readability based on the number of words per sentence and syllables per word [30,31]. The Flesch readability score ranges from 0 to 100, with 0 being extremely difficult and 100 being extremely easy to read. Grade levels range from 1 to 12; grade 1 is the easiest, and grade 12 is the hardest to read. The characters and word counts of the text messages were also calculated.

## Results

### Step 1: Co-design Workshop

Consumers (n=2) and health professionals and researchers (n=4; health psychologist, specialist breast cancer surgeon, physiotherapist and digital health researcher, and psychology researcher) attended the workshop. The group decided that the program would be one-way (no replies) as consumers did not want to feel pressure to reply. Our team’s previous research also showed that one-way messaging was well-liked by patients with heart disease and helped them feel supported [27]. Next, the group decided that the text messages would be delivered randomly 4 times per week, as consumers felt that 1-2 messages were too few to be useful but 6 or 7 would be annoying. In our previous research, 4 messages per week were found to be acceptable [27]. Unlike our team’s previous research, which only delivered messages on weekdays [14], the consumers desired messages to be delivered Monday-Saturday (maximum 1 message per day) because people may have more time to read messages on the weekend. Messages would be delivered at 9 AM, noon, 3 PM, or 6 PM to mimic the unpredictability of how a friend or family member would text and gain insights into future process evaluations as to what times are best. The team decided that 1 message per theme would be delivered per week in random order, as consumers indicated that a specific order was not required. The co-designed message content themes were (1) physical activity and healthy eating, (2) endocrine therapy tablet (medication) adherence and side effects, (3) social and emotional well-being, and (4) general breast cancer information. Moreover, participants wanted messages to contain links to trustworthy websites because it can be challenging to navigate cancer information on the web. It was also agreed that the program would be semipersonalized according to the participants’ preferred name or *nickname*, and there would be specific messages for participants taking endocrine therapy tablets because these tablets can cause challenging side effects and medication nonadherence [32].

A total of 274 messages were co-designed; 157 messages were drafted during the workshop, and 117 were created by the authors, including the citizen collaborator, after the workshop. The citizen collaborator’s content subtheme ideas included posttreatment financial support, difficulties returning to work (fatigue, treatment side effects, or cognitive load), and practical solutions for medication side effects. After revision of the messages by authors AS, JR, SRP, and RR, 189 co-designed messages remained for evaluation in step 2. Reasons for deletion included repetitive ideas or content (65/274, 24%), negative message tone (11/274, 4%), and message content not being relevant to breast cancer survivors (9/274, 4%)

### Step 2: Text Message Content Evaluation

#### Participants

Consumers (14/16, 87.5%) and health professionals or researchers (14/14, 100%) completed the message evaluation; 2 consumers (2/16, 12.5%) did not return their surveys and were not included in the analyses. The mean age of consumers was 60 (SD 10) years, and the mean age of health professionals or researchers was 46 (SD 8) years. Most participants were White (12/14, 86% of consumers; 12/14, 86% of health professionals or researchers) women (14/14, 100% of consumers; 12/14, 86% of health professionals or researchers).

#### Consumer and Health Professional Evaluation

The 189 co-designed text messages were evaluated 870 times: 443 times by consumers, and 427 times by health professionals or researchers (mean 2.34 consumer and 2.26 health professional evaluations per message). Overall, participants *agreed* or *strongly agreed* that the messages were clear (799/870, 91.8%), useful (746/870, 85.7%), and appropriate (732/870, 84.1%). Most messages (156/189, 82.5%) received a mean score of ≥12 out of 15 from consumers (153/189, 80.9%) and health professionals (156/189, 82.5%). However, 50 messages received a different mean score from consumers versus health professionals or researchers; 25 were evaluated highly by consumers (mean score ≥12/15, 80%) but not health professionals or researchers, and the other 25 were evaluated poorly by consumers (mean score <12/15, 80%). Examples of discrepant evaluation scores and free-text feedback are presented in Table 2. The number of free-text responses was similar between health professionals (n=150) and consumers (n=156), suggesting that the media on which they conducted the surveys (in person vs on the web) did not bias the feedback. Free-text feedback themes from health professionals or researchers (n=14) focused on grammar, technical terms, and adding web links. Consumers’ (n=14) free-text feedback focused on personal experiences and increased positivity in messages.

**Table 2:** Example text messages and decisions based on discrepant consumers’ versus health professionals and researchers’ mean ratings (out of 15) and supporting feedback quotes.

Message themes and subthemes					Decision
**General breast cancer information**					
	**Returning to work**				
		“Hi [preferred name], returning to work can be difficult - some women have found it helpful to openly discussing their needs and working arrangements with their managers so that everyone is on the same page”	Consumers’ mean rating 10/15 (67%): “Work were not helpful and sick leave all used up - refused flexible work and had to feel guilty. Head of [Human Resources] however was supportive, not line manager and above” (female, age 65 years) Health professional and researchers’ mean rating 12/15 (80%): “wording change after managers ‘discussing’ to discuss eg: ‘so a plan can be made’ or just stop after managers” (female, age 62 years)		Deleted
	**Link to web-based breast cancer fact sheets or resources**				
		“Did you know that the Breast Cancer Network Australia provides a variety of kits, booklets and fact sheets where you can find more information? Check it out: [insert link]”	Consumers’ mean rating 13.5/15 (90%): “helpful” (female, age 65 years) Health professionals and researchers’ mean rating 11/15 (73%): “All patients with breast cancer get this information at the time of their diagnosis” (female, age 54 years)		Edited
**Medication adherence and side effects**					
	**Hot flushes**				
		“If you are experiencing hot flushes at night, give a cool shower right before bed a go and see if that helps!”	Consumers’ mean rating 12/15 (80%): “aware this is a side effect especially of hormonal treatment” (female, age 65 years) Health professionals and researchers’ mean rating 10/15 (67%): “‘Have a cold shower’ I’m not sure that cold showers will be popular??” (Male, age 51 years); “‘Wording not good – suggest’...taking a cool shower just before going to bed might help’ or something similar” (female, age 54 years)		Deleted
**Physical activity**					
	**Counting steps**				
		“Apps, watches or other step counters can help with tracking your steps each day - The World Health Organisation recommends 10,000 steps per day. How many do you usually do [preferred name]?”	Consumers’ mean rating 11.5/15 (77%): “While the message is clear, I don’t think I need to receive it. Messages like these are not personal and supportive. Just that not everyone has apps, younger generation like my daughter does, with her fitbit she tracks steps. I don’t have one. A message like 30 mins (walking) of exercise 3-5x a week is recommended, how often do you exercise” (female, age 45 years) Health professionals and researchers’ mean rating 15/15 (100%): “should there be a link [or] reasoning why useful for [Breast Cancer Institute] patient” (female, age 38 years)		Deleted. Consumer’s suggestion is referring to another message she evaluated, which is included in the final message bank.
**Nutrition**					
	**Portion sizes**				
		“Hey [preferred name], some people eat too much of a good thing. As a general rule - it's good to keep portion sizes around the size of your fist”	Consumers’ mean rating 13.5/15 (90%): “Good information” (female, age 50 years) Health professional and researchers’ mean rating 11.5/15 (77%): “Could delete. Fruit and veg portions is larger than fist” (female, age 31 years)		Edited
**Social and emotional well-being**					
	**Setting goals for self-care**				
		“Hi [preferred name], setting aside time for yourself in your diary or on your phone can make it easier to stick to - whether it’s a walk, yoga or just reading a good book, treat yourself to some 'you' time”	Consumers’ mean rating 12.33/15 (82%): “resources need to be more available. [they are] not always in a prominent place. These resources would have helped - had to really search.” (female, age 65 years) Health professionals and researchers’ mean rating 10/15 (67%): “This message seems too busy. I would simplify. Maybe make into 2 messages as you are tapping into 2 different ideas: regular practice and ‘me time’. I would separate.” (female, age 55 years)		Edited

Oral interview feedback from consumers (n=3) and free-text feedback from the citizen collaborator revealed three important themes for consideration in message refinement: prevention of loneliness, impacts of treatment and medication side effects, and motivation to exercise. Example quotes for each theme are presented in Textbox 1. Feedback also revealed that the term *journey* was polarizing. For example, a consumer stated, “Many women don’t like the word journey. Could use breast cancer experiences instead” (female, age 41 years), and a health professional’s feedback mirrored this sentiment: “some people don’t like the word journey, maybe consider alternative eg experience” (female, age 62 years). Therefore, *journey* was replaced with the term *experience* throughout. Consumers also felt that messages relating to alcohol and cooking food on a barbeque were not appropriate in some cultures. For example, a consumer stated, “I heard the BBQ is not good for cancer people because when protein and oil getting burnt...burnt food is no good for cancer” and later explained, “here is one about limit[ing] alcohol. It’s a good message but some people when they read that message, even they don’t want to drink alcohol. Some people read it and they might think ‘oh maybe I can drink alcohol’” (consumer, female, age 50 years). All messages about alcohol were deleted, and the word *BBQ* was replaced with a suggestion to *bake* the food.

**Consumer and citizen collaborator message content themes and quotes.**

**Theme 1: Prevention of Loneliness and Isolation**

The messages [that] give information, give some encouragement because after treatment, I stayed home and I felt very lonely.

Consumer, female, age 50 years

**Theme 2: Impact of Side Effects**

I was slowly recuperating pretty good, but I’m still not over it yet, it’s been over 12 months...Tiredness, sometimes I can’t do housework.

Consumer, female, age 76 years

Fatigue was huge. And it would always hit you sort of 2, 3 o’clock in the afternoon.

Consumer, female, age 56 years

**Theme 3: Motivation for Exercise**

have an exercise buddy or just a friend that you meet to go and have a walk with or walk your dog...just go I’m meeting so and so and we’re going to do this today, and it’s like keeping an appointment

Consumer, female, age 56 years

[My smartwatch] counted the amount of steps I did every day...so it would tell me how many kilometres I had done. It gives you this sense of achievement at the end of the week.

Consumer, female, age 56 years

One thing you could add is a reminder that finding time to exercise and eat healthy can be really hard when you struggle with fatigue, but that it will actually help give you more energy.

Citizen collaborator, female, age 30 years

after operation...it’s been 3 years...but still pain and the scar is hard. When I’m moving it feels hard [tight]. The doctor said more exercises, stretch and do some physical therapy.

Consumer, female, age 50 years

On the basis of the feedback (Likert scale, free-text questions, and interviews), 36.5% (69/189) of messages were edited, 37.6% (71/189) were deleted (score <12 or repetitive concept), and 12 new messages were created; 130 evidence-based, co-designed messages remained: 55.4% (72/130) of messages relating to the theme of physical activity and healthy eating, 29.2% (38/130) regarding social and emotional well-being, 32.3% (42/130) regarding general breast cancer information, and 28.5% (37/130) regarding medication adherence and side effects. Subthemes from step 1, such as financial support and practical solutions for side effects (fatigue, hot flushes, and cognitive load), were included within these four broader themes.

#### Readability

The 130 text messages had a mean Flesch-Kincaid grade level of 7.12 (SD 2.84) and an ease-of-reading score of 68.92 (SD 15.46), representing *standard* reading ease, which means that the texts will be easy to read for anyone with grade (or year) 7 education and higher [31]. The text messages had a mean of 2.02 (SD 0.69) sentences, 29.09 (SD 6.72) words, or 132.74 (SD 31.69) characters.

## Discussion

### Principal Findings

This study reports the outcomes of a co-design process [14] and the development of a bank of evidence-based text messages. To our knowledge, this is the first SMS text message intervention to be co-designed by breast cancer survivors, a citizen collaborator, health professionals, and researchers, and includes both mental and physical health messages. The message content was combined with clinical guidelines [28] and behavior change techniques [23]. Messages were evaluated highly for ease of understanding, usefulness, and appropriateness for breast cancer survivors by consumers, health professionals, and researchers. Although there were discrepancies between some consumers’ and health professionals and researchers’ mean message ratings, the consumers’ feedback was prioritized to ensure that their voices were at the forefront of the co-design process. Compared with the initial bank of draft text messages (n=274), the resulting 130 evidence-based co-designed text messages were evaluated highly (score ≥12/15, 80% and positive qualitative feedback) by participants and focused on topics of interest to consumers—namely, social and emotional well-being, healthy eating, physical activity, medication adherence, side effects, and general breast cancer information. Overall, this iterative co-design process was feasible, produced an improved final text message bank, and highlighted the importance of consumer co-design.

SMS text message interventions are becoming increasingly popular as mHealth strategies [33]. However, there is sparse research on women with breast cancer [16-21], and few programs describe the process of text message content development [34]. For example, health literacy is often overlooked or not reported in the development of eHealth programs [35]. In this study, messages were short and rated highly for ease of understanding, which is supported by the Flesch-Kincaid grade level 7 and readability score (SD 68.9). These scores indicate that anyone with an education grade of 7 or above would likely understand the messages [31], which may reduce health literacy barriers [30] and increase user acceptability [27]. Moreover, health professionals, researchers, and consumers feedback revealed that a positive message tone was important, which is consistent across studies [27,36]. Recent systematic reviews reveal that message tailoring and personalization may [37] or may not [38] improve program efficacy, and message content does not need to be based on a theoretical framework to be effective [38]. Qualitative results suggest that it may be the overall impact of the program rather than a single program component that end users find helpful [27]. However, for adequate comparison across interventions, the development of message content should be clearly reported [37,38]. Factors including the co-design strategy, behavior change theories, readability, and message personalization can help elucidate what makes an effective and well-liked intervention.

This study also provides a framework for developing and implementing SMS text message programs with consumers’ voices at the forefront. Previous studies have developed and refined messages with health professionals, and consumers reviewed the edited messages [14,39]. This strategy may miss message topics or lived experiences important to consumers and limit program and delivery impact. Conversely, this study simultaneously involved consumers and a citizen collaborator with health professionals and researchers throughout the message development, review, and refinement. Moreover, all participants (consumers, a citizen collaborator, health professionals, and researchers) reviewed the same initial messages and were provided equal opportunity for opinions; furthermore, we prioritized consumer ratings when refining messages. Research shows that developing programs with consumers and citizen collaborators can improve user acceptance and engagement [7,9], which may explain the high rating of message usefulness and acceptability from both consumers and health professionals and researchers in this study. However, an ongoing challenge is to provide adequate compensation to consumers for their involvement and efforts. The National Health and Medical Research Council recently released guidelines for the involvement of consumers in research, which suggest that consumer reimbursement (financial, authorship, or acknowledgment) should be considered when planning a project or applying for grants [40]. These guidelines may help future researchers to identify barriers and enablers in consumer co-design.

### Implications for Practice

The proliferation of breast cancer survivors is a testament to improvement in treatment [1]. However, women’s mental health can be impacted years after a breast cancer diagnosis [41,42] and increase the urgency for ongoing supportive care. There is evidence that eHealth self-management programs *during* cancer treatment can reduce women’s distress and depression [43,44]. However, few programs address the mental health concerns that arise *after* treatment [45]. In fact, many text message programs focus on improving physical behaviors such as physical activity, healthy eating, smoking cessation, and medication adherence [34]. However, previous research shows that mental and physical health are closely related and that consumers desire mental health information [5,46,47]. This program endeavored to fill this gap by co-designing messages for mental health after breast cancer treatment. The consumer feedback from this study provided further evidence that loneliness and treatment side effects are ongoing concerns that may impact mental health. This world-first co-designed program for breast cancer survivors may therefore add to current posttreatment support services by offering an overall lifestyle-focused approach, including both mental and physical health promotion.

Although the study provides new evidence for the co-design of SMS text message interventions for breast cancer survivors, it has some limitations. The number of consumers was relatively small, and most were White, which may not be generalizable to the priorities of the wider breast cancer community. However, cultural considerations, such as alcohol intake and cooking styles, were considered in the message evaluations and refinements. Consumers were recruited from WBCI, which services a large (about 2.3 million) multilingual and socioeconomically diverse population in Western Sydney. Half (49%) of the Western Sydney population speak a non-English language at home, including 8.5% who reported speaking English *not well* or *not at all* [22]. It is possible that the requirement to read and understand English limited some people from participating. However, this study demonstrates that the co-design process is acceptable and can be easily replicated in other populations or languages. To increase the diversity and number of message reviewers, future research should provide videoconference options for co-design workshops and web-based options for text message evaluation surveys, where possible and allowable through ethics. Reducing access barriers will allow women from distant or rural communities to offer important inputs. Moreover, the final text message bank will be evaluated in a randomized controlled trial and process evaluation [48]. A diverse sample of 160 women will be recruited to provide feedback via a questionnaire and focus groups to elucidate the feasibility and acceptability of the message content and overall one-way program within the wider breast cancer community.

### Conclusions

Consumers, a citizen collaborator, health professionals, and researchers successfully co-designed a set of 130 evidence-based lifestyle-focused text messages to support women’s health after breast cancer treatment. The co-design process resulted in an improved final bank of text messages and highlighted the importance of involving consumers as equal co-designers. This process can easily be replicated to develop SMS text message interventions for other patient populations.

## Abbreviations

mHealth: mobile health
WBCI: Westmead Breast Cancer Institute
